# PABPC1 Restricts Bat-Origin Swine Acute Diarrhea Syndrome Coronavirus Infection via TOLLIP-Mediated Degradation of Viral Nucleocapsid Protein

**DOI:** 10.3390/pathogens15070752

**Published:** 2026-07-17

**Authors:** Maowen Sun, Cong Yuan, Yu Zhang, Xueliang Zhu, Lei Shi, Yueyue Duan, Wenquan Mao, Luyao Li, Yanghe Liu, Qi Wang

**Affiliations:** State Key Laboratory of Animal Disease Control and Prevention, Lanzhou Veterinary Research Institute, Chinese Academy of Agricultural Sciences, Lanzhou 730046, China

**Keywords:** swine acute diarrhea syndrome coronavirus, PABPC1, host restriction factor, TOLLIP, autophagy

## Abstract

The emergence of swine acute diarrhea syndrome coronavirus (SADS-CoV), an alpha-coronavirus that causes fatal enteric disease in neonatal piglets with mortality rates up to 90%, demonstrates that bat-origin coronavirus has expanded its host range to pigs. Although SADS-CoV exhibits significant pandemic potential and capacity for cross-species transmission, the replication mechanisms of SADS-CoV remain largely unexplored. Identifying host factors responsible for SADS-CoV replication and elucidating its underlying mechanisms is essential for advancing fundamental knowledge of coronavirus biology and developing antiviral therapies. Here, we identified PABPC1 as a novel interactor of the SADS-CoV nucleocapsid (N) protein. Overexpression of PABPC1 restricted SADS-CoV infection, whereas knockdown of PABPC1 enhanced viral replication. Further study indicated PABPC1 as a host restriction factor of SADS-CoV in a manner dependent on its PABC domain. Mechanistically, PABPC1 enhances the ubiquitination of the N protein, therefore facilitating its recognition by the cargo receptor TOLLIP for selective autophagic degradation. This study systematically analyzes the interaction of host factors and the SADS-CoV N protein and identifies PABPC1 as a host restriction factor that limits viral replication via TOLLIP-mediated selective autophagy degradation of the N protein. These findings expand our knowledge of the SADS-CoV replication mechanism and provide additional antiviral strategies for controlling SADS-CoV.

## 1. Introduction

Coronaviruses (CoVs) have the largest genome of all known RNA viruses and have caused three global pandemics in humans. Coronaviruses belong to the order of *Nidovirales*, family of *Coronaviridae*, and subfamily of *Orthocoronavirinae*, which are classified into four genera based on viral genomic structure and phylogenetic relationships: *Alphacoronaviruses* (*α*-CoVs), *Betacoronaviruses* (*β*-CoVs), *Gammacoronaviruses* (γ-CoVs), and *Deltacoronaviruses* (δ-CoVs) [1]. From 2016 to 2017, a fatal swine acute diarrhea syndrome coronavirus (SADS-CoV) emerged in Guangdong province [2,3]. SADS-CoV, which is a bat-origin *α*-CoV, has symptoms indistinguishable from porcine epidemic diarrhea virus (PEDV) and transmissible gastroenteritis virus (TGEV), and results in 90% mortality in piglets under 5 days old [2,3]. Unlike the known coronaviruses, SADS-CoV infection is independent of the well-characterized coronavirus receptors such as ACE2, DPP4, CEACAM1, or APN [4,5]. However, more than 20 cell lines derived from different species, including humans, pigs, bats, and mice, as well as sucking BALB/c or C57BL/6J mice, are susceptible to SADS-CoV infection [4,6,7,8]. A few human coronaviruses that originated from bats have resulted in respiratory illnesses and even fatal disease. No SADS-CoV infection has been reported in humans, but there is a high risk of spillover to humans because of its broad-spectrum cell tropism and close contact with pigs [3,4,5,6]. Therefore, the potential risk of SADS-CoV for cross-species transmission should not be ignored [4,6,7,8]. Notably, SADS-related coronavirus (SADSr-CoV) detected in bats from 2013 to 2016 shares high sequence similarity with SADS-CoV and has shown potential for interspecies infection [3,9]. There is currently no licensed vaccine or therapeutic against SADS-CoV and SADSr-CoVs. Therefore, it is imperative to improve prevention and control strategies for these viruses by understanding the mechanisms underlying virus–host interaction.

The nucleocapsid (N) protein is an abundant structural protein of CoVs, characterized by high conservation, low mutability, and strong affinity for viral RNA. It forms a ribonucleoprotein (RNP) complex with the viral RNA, enhances viral RNA transcription efficiency, suppresses host innate immune response, and alters host biological processes [10,11]. The coronavirus N protein has also been considered as a promising antiviral target [11]. A comprehensive understanding of the interactions between the SADS-CoV N protein and host factors will not only advance our knowledge of SADS-CoV pathogenesis but also provide new insights for developing therapeutic strategies against other bat-origin SADSr-CoVs. Poly (A)-binding proteins (PABPs) are highly conserved RNA-binding proteins (RBPs) that bind the 3′ poly (A) tail of mRNA [12]. Cytoplasmic PABPs, known as PABPCs, stabilize mRNA by interacting with its poly (A) tail and enhance translation initiation through recruitment of translation initiation factors to form a translation initiation complex. In contrast, nuclear PABPs are associated with poly (A) tail synthesis and mRNA maturation [13]. Poly (A)-binding protein cytoplasmic 1 (PABPC1), a member of the PABPC family, regulates the translation, deadenylation, and stability of mRNA [14,15]. In addition to its role in mRNA regulation, PABPC1 is considered a core component of stress granules (SGs), exhibiting antiviral activity [16,17,18,19,20]. However, the role of PABPC1 in virus infection remains controversial. Previous reports indicate that ectopic expression of PABPC1 exerts antiviral activity against porcine epidemic diarrhea virus (PEDV), picornaviruses, and pneumoviruses [17,18,19,20]. Conversely, other evidence demonstrates that PABPC1 either promotes the translation of PEDV mRNA or, together with translation initiation factor eIF4F, is captured by the viral N protein, thereby facilitating PEDV replication [21,22]. However, the role of PABPC1 in SADS-CoV replication is uncharacterized.

In this study, we identified host factors interacting with the SADS-CoV N protein using immunoprecipitation coupled with mass spectrometry (IP-MS) and confirmed that PABPC1 interacts with the SADS-CoV N protein. Notably, PABPC1 exhibited significant antiviral activity against SADS-CoV infection, while knockdown of PABPC1 facilitated SADS-CoV replication. Mechanistically, PABPC1 binds to the SADS-CoV N protein and promotes its degradation through the selective autophagy pathway. Subsequent research confirmed that PABPC1 enhances the ubiquitination of the SADS-CoV N protein, which is further recognized by the cargo receptor TOLLIP, resulting in selective autophagic degradation of the N protein. This study contributes to our understanding of the host–SADS-CoV interaction mechanisms and highlights the potential of employing either PABPC1 or its analogs as antiviral strategies against SADS-CoV.

## 2. Materials and Methods

### 2.1. Cells and Virus

HEK293T and Huh7 cells were acquired from ATCC and stored in our laboratory. Both HEK293T and Huh7 cells were cultured in Dulbecco’s Modified Eagle Medium (DMEM) (Gibco, CA, USA) supplemented with 10% fetal bovine serum (FBS) (VivaCell, Shanghai, China) and 2% penicillin–streptomycin solution (BI, HaZafon, Israel). The SADS-CoV GD04 strain (GenBank: MF167434.1) was kindly provided by Yong-chang Cao [23] and stored at −80 °C in our laboratory.

### 2.2. Plasmids and Antibodies

The SADS-CoV *N* gene was successfully cloned into the p3xFlag-CMV-10 vector in our laboratory [24]. The pCMV-HA-Ub was stored in our laboratory. The *PABPC1* genes were amplified from cDNA of Huh7 cells and subsequently cloned into the pCAGGS-HA vector or plvx-IRES-puro vector, named as pHA-PABPC1 or pHis-PABPC1, respectively. The PABPC1 truncated mutants were amplified from pHA-PABPC1 and inserted into the pCAGGS-HA vector. All primers used for the construction of the plasmids are listed in Table 1. The Flag-tagged cargo receptors, including NDP52, P62/SQSTM1, TOLLIP, and OPTN, were purchased from MailGene Biotech (Beijing, China). Rabbit anti-SADS-CoV N protein was produced in-house. The antibodies against DYKDDDDK (66008-4-Ig), GAPDH (10494-1-AP), HA (51064-2-AP), His (66005-1-Ig), p62/SQSTM1 (66184-1-Ig), TOLLIP (11315-1-AP), and PABPC1 (10970-1-AP) were obtained from Proteintech (Wuhan, China). Goat anti-mouse IgG H&L (HRP) (ab205719) and goat anti-rabbit IgG H&L (HRP) (ab97051) were purchased from Abcam (Cambridge, UK). Goat anti-rabbit IgG (H + L) Alexa Fluor 488 (A-11008) and goat anti-mouse IgG (H + L) cross-adsorbed secondary antibody Alexa Fluor 594 (A-11005) were purchased from ThermoFisher Scientific (Waltham, MA, USA).

### 2.3. Western Blotting

Cells were lysed at 4 °C for 15 min using NP40 lysis buffer (Beyotime, Shanghai, China) supplemented with 1% PMSF (Solarbio, Beijing, China). The lysates were then centrifuged at 4 °C for 15 min. Total proteins were boiled in 5× SDS-PAGE loading buffer (Biosharp, Hefei, China) for 10 min. After separation by SADS-PAGE, proteins were transferred onto a nitrocellulose filter (NC) membrane (Pall, Port Washington, NY, USA). The NC membrane was blocked with 5% Difco^TM^ Skim Milk (BD, Franklin Lakes, NJ, USA) for 2 h at room temperature, followed by incubation with the indicated antibody overnight at 4 °C. The membrane was washed three times using TBST buffer and subsequently incubated with HRP-conjugated secondary antibodies for 1 h at room temperature. After washing with TBST buffer three times, protein signals were measured by incubating the NC membrane with the WesternBright ECL kit (Advansta, CA, USA) and visualized using a chemiluminescence imaging system.

### 2.4. Co-Immunoprecipitation (Co-IP) Assay

The plasmids of HA-PABPC1 or Flag-N, individually or together, were transfected into HEK293T cells using PEI transfection reagent. After 24 h post-transfection (hpt), cells were lysed using NP40 lysis buffer supplemented with 1% PMSF at 4 °C for 15 min. The lysate was then incubated overnight at 4 °C with Flag-beads (Merck, Darmstadt, Germany). Following incubation, the Flag-beads were washed five times using NP40 lysis buffer and subjected to Western blotting using rabbit antibodies against Flag, HA, and GAPDH.

### 2.5. Liquid Chromatography–Mass Spectrometry (LC-MS) Analysis

Huh7 cells were transfected with either pFlag-N or an empty vector using jetPRIME transfection reagent (Polyplus, Strasbourg, France). After 24 hpt, the supernatants of cell lysates were collected and immunoprecipitated using Flag-beads as described in the Co-IP assay. The immunoprecipitated complexes were separated by SADS-PAGE and subsequently stained with Coomassie blue. Protein bands uniquely present in the N-enriched samples were cut and destained in 50 mM NH_4_HCO_3_ in 50% acetonitrile until clear. Gel pieces were treated with trypsin overnight at 37 °C. The peptides were extracted, dissolved, and analyzed by LC-MS. The LC-MS data were processed using Proteome Discoverer 1.3. All LC-MS analyses were processed at PTM BIO (Hangzhou, China).

### 2.6. Indirect Fluorescent Assay (IFA)

Huh7 cells were transfected with pHA-PABPC1 or empty vector using jetPRIME transfection reagent. After 24 hpt, cells were infected with SADS-CoV at a multiplicity of infection (MOI) of 1 for 24 h. The cells were treated with 4% paraformaldehyde for 20 min at room temperature, followed by three times using PBS. After permeabilization with 0.3% Triton X-100 for 15 min at room temperature, cells were washed using PBS three times and incubated for 2 h at 37 °C with a mouse antibody against HA and a rabbit antibody against the SADS-CoV N protein. Following three PBS washes, cells were incubated with goat anti-rabbit IgG (H + L) Alexa Fluor^TM^ 488 and goat anti-mouse IgG (H + L) Alexa Fluor 594 for 45 min at room temperature. After a final round of PBS washes, nuclei were stained with DAPI (Solarbio, Beijing, China) for 10 min. Images were captured by an inverted fluorescence microscope.

### 2.7. Laser Confocal Microscopy

Vero cells were transfected with the indicated plasmids using jetPRIME transfection reagent. Subsequently, cells were fixed and permeabilized using the indicated reagents, followed by three washes with PBS as described in the protocol of IFA. The cells were incubated with mouse anti-Flag and rabbit anti-HA for 2 h at 37 °C and washed with PBS. Following this, the cells were incubated with goat anti-mouse IgG (H + L) Alexa Fluor 594 or goat anti-rabbit IgG (H + L) Alexa Fluor 488 for 45 min at room temperature. Nuclei were stained with DAPI for 10 min. The cellular distributions of the SADS-CoV N protein and PABPC1 were observed by confocal microscopy.

### 2.8. Reverse-Transcription Quantitative PCR (RT-qPCR)

Huh7 cells were seeded in a 24-well plate and transfected with pHA-PABPC1 or empty vector using jetPRIMER transfection reagent for 24 h, followed by infection with SADS-CoV at an MOI of 1. After 24 h post-infection (hpi), total RNA was extracted using Total RNA Kit I (Omega, Norcross, GA, USA) according to the manufacturer’s instructions and reverse-transcribed into cDNA by PrimeScrip RT Master Mix (Perfect Real Time) (Takara, Kyoto, Japan). The cDNAs from Huh7 cells were used as templates to determine viral genomic copy number by quantitative PCR. The primer sequences used in this study are listed in Table 1.

### 2.9. RNA Interference Assay

To knock down PABPC1, siRNA targeting human PABPC1 (siPABPC1) (5′-GCUCCUAAAU-GAUCGCAAATT-3′) was transfected into Huh7 cells using jetPRIME transfection reagent according to the manufacturer’s instructions for 24 h, followed by infection with SADS-CoV at an MOI of 0.1 for 24 h. The SADS-CoV replication efficiency was determined by Western blotting or TCID50.

### 2.10. Inhibitor Treatment Assay

HEK293T cells were cotransfected with pFlag-SADS-CoV N and either pHA-PABPC1 or an empty vector. After 24 hpt, cells were treated with the autophagosome inhibitors 3-Methyladenine (HY-19312) and Bafilomycin A1 (HY-100558) at 5 mM and 100 nM, respectively, or with the proteasome inhibitor MG132 (HY-13259) at 20 µM for 8 h. And then the expression of protein was determined by Western blotting using the indicated antibodies.

### 2.11. CRISPR/Cas9 Knockout

SgRNAs targeting p62/SQSTM1 (5′-GAAGATGTCATCCTTCACGT-3′) or TOLLIP (5′-GGGCCGACTGAACATCACGG-3′) were synthesized by Tsingke Biotech (Beijing, China) and subsequently cloned into the lentiCRISPR V2 vector. To produce lentiviruses, the plasmid of lentiCRISPR V2 harboring sgRNA of p62/SQSTM1 or TOLLIP was cotransfected with other helper plasmids into HEK293T cells using the PEI transfection reagent. After 48 hpt, the cell supernatant was collected and filtered with a 0.45 µM filter. HEK293T or Huh7 cells were then infected with the lentiviruses. After 48 hpi, the cells were cultured with DMEM supplemented with 10% FBS and puromycin at 1 µg/mL. One week later, surviving cells were cultured. TOLLIP or P62 knockout cells were validated by Western blotting using anti-TOLLIP or P62 antibody, respectively.

### 2.12. Statistical Analysis

All experiments in the study were performed in triplicate. Data were analyzed using GraphPad Prism 8.0.2. *p* > 0.05, < 0.05, 0.01, or 0.001 were represented by no significant difference (ns), significant difference (*), highly significant difference (**), or extremely significant difference (***), respectively.

## 3. Results

### 3.1. PABPC1 Interacts with the SADS-CoV Nucleocapsid Protein

**Figure 1:**
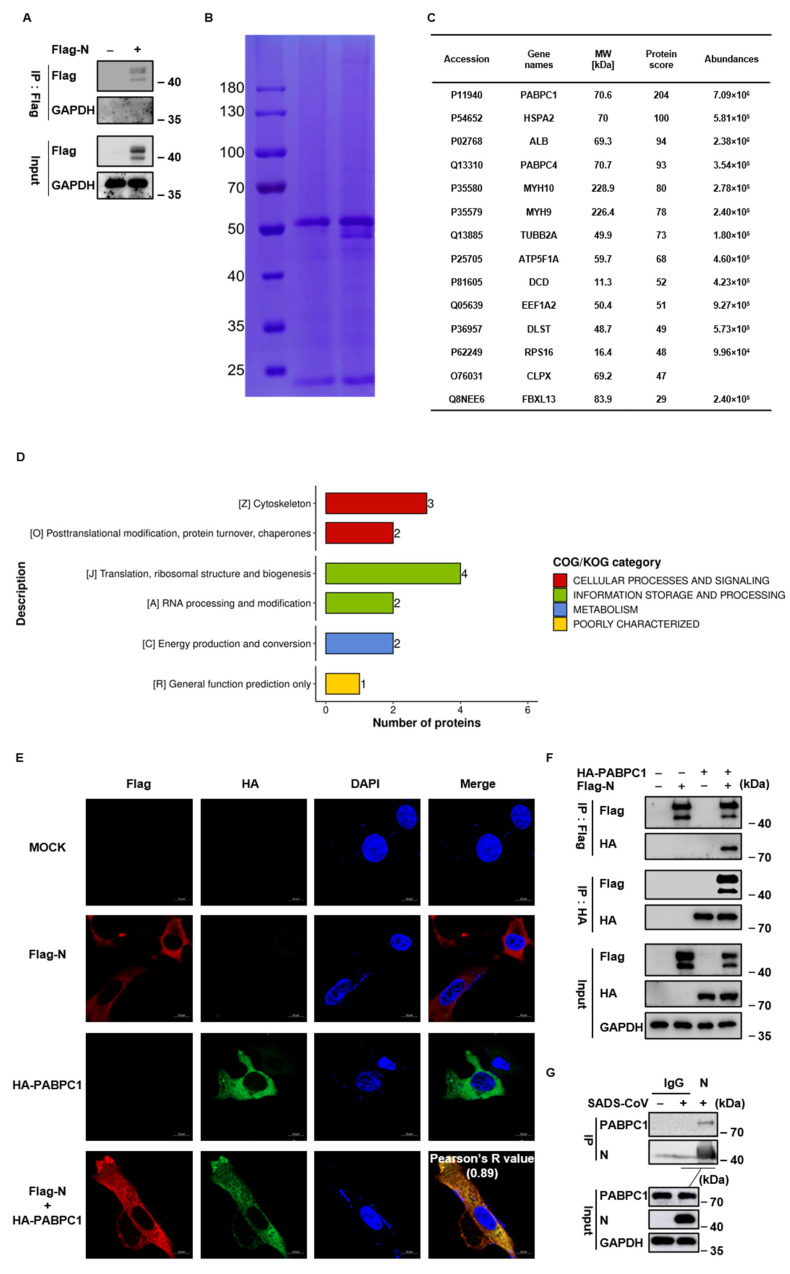
Identification of the host factors that interact with the SADS-CoV N protein. (**A**,**B**) pFlag-N or empty vector was transfected into Huh7 cells. Cell lysates were immunoprecipitated using Flag-beads, followed by SDS-PAGE separation and analysis by Western blotting (**A**) and Coomassie staining (**B**). (**C**) The candidate host factors were identified through IP-MS analysis. (**D**) The functional classification of the potential interactors of the N protein was performed using COG analysis. (**E**) The colocalization of the N protein with PABPC1 was assessed in Vero cells using laser confocal microscopy. Scale bar, 10 µm. (**F**) The plasmids of pHA-PABPC1 and pFlag-N were cotransfected into HEK293T cells. Cell lysates were immunoprecipitated with Flag-beads or HA-beads and followed by Western blotting analysis using antibodies against Flag, HA, and GAPDH, respectively. (**G**) Huh7 cells were infected with SADS-CoV at an MOI of 1 for 24 h. The cell lysates were used for a co-immunoprecipitation (Co-IP) assay to detect the interaction between endogenous PABPC1 and the SADS-CoV N protein. GAPDH was used as a loading control.

The nucleocapsid (N) protein, the most abundant protein of coronaviruses, is a multifunctional protein that plays a critical role in immune evasion, virion packaging, and viral RNA synthesis. To systematically analyze and identify the host protein interacting with the SADS-CoV N protein, the plasmid of Flag-tagged empty vector or N was transfected into Huh7 cells, followed by the N protein complexes being affinity-purified using Flag-beads and analyzed by mass spectrometry at 24 h post-transfection (hpt) (Figure 1A). The precipitated proteins were separated by SDS-PAGE and processed by Coomassie blue staining (Figure 1B). Compared with the empty vector control, several distinct and specific bands at about 40–50 kDa, 70–100 kDa, and above 180 kDa were observed in immunoprecipitates from the N-transfected cells. These bands were subsequently identified by liquid chromatography–mass spectrometry (LC-MS) (Figure 1B). Among the fourteen identified proteins that potentially interact with the SADS-CoV N protein (Figure 1C), these proteins, based on their functions, were classed into cytoskeleton (MYH9, MYH10, and TUBB2A); posttranslational modification, protein turnover, and chaperones (HSPA2 and CLPX); translation, ribosomal structure and biogenesis (PABPC1, PABPC4, EEF1A2, and RPS16); RNA processing and modification (PABPC1 and PABPC4); energy production and conversion (ATP5F1A and DLST); and general function prediction only (FBXL13) (Figure 1D). PABPC4, a known interactor of the SADS-CoV N protein, was identified in our analysis. Notably, PABPC1, a homologue of PABPC4, was also identified and ranked at the top among these proteins (Figure 1C). To further validate the IP-MS results, we determined the interaction between N and PABPC1 using an indirect fluorescent assay (IFA) and a co-immunoprecipitation (Co-IP) assay. The subcellular localization of PABPC1 and N was determined in Vero cells that were cotransfected with pFlag-N and pHA-PABPC1. A significant colocalization of N protein and PABPC1 was observed in the cytoplasm (Figure 1E). After cotransfection of pHA-PABPC1 with either pFlag-N or an empty vector together or individually in HEK293T cells, we detected a specific interaction of N protein with PABPC1 using HA- or Flag-beads (Figure 1F). The SADS-CoV N protein successfully immunoprecipitated with endogenous PABPC1 in SADS-CoV-infected Huh7 cells (Figure 1G). Both PABPC1 and N protein have a high affinity for RNA; we thus explored the interaction of PABPC1 and N by treatment with RNase. The interaction of PABPC1 and N was significantly abrogated in the presence of RNase (Figure S1A), indicating that cellular RNA is necessary for their interaction. Together, these results confirmed that PABPC1 is a novel interactor of the SADS-CoV N protein.

### 3.2. PABPC1 Inhibits SADS-CoV Replication

**Figure 2:**
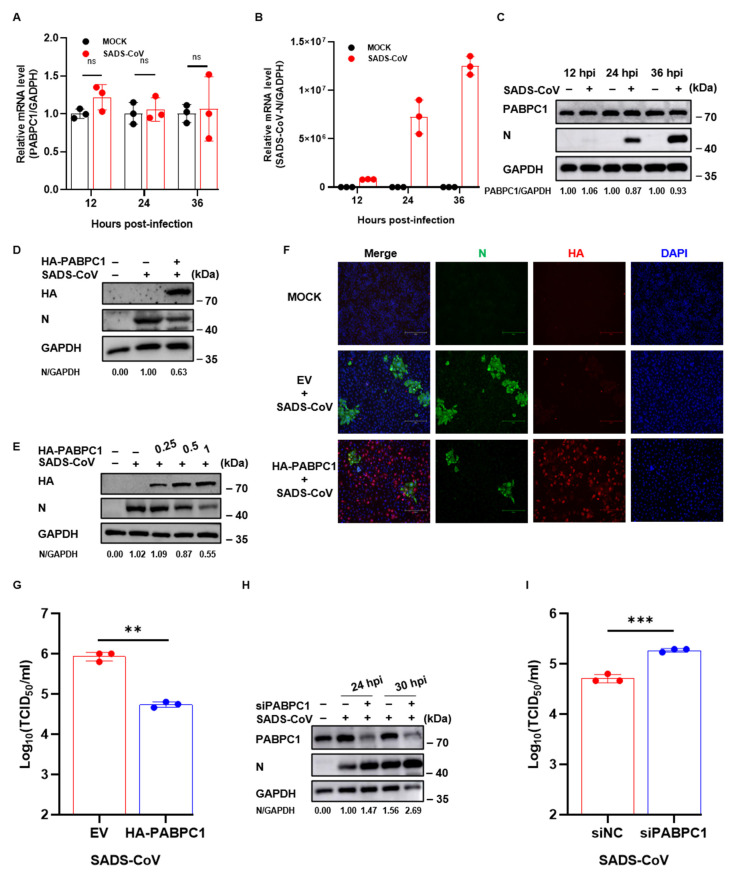
PABPC1 inhibits SADS-CoV replication. (**A**,**B**) Huh7 cells were infected with SADS-CoV at an MOI of 1. The transcription level of the *PABPC1* (**A**) or *N* (**B**) gene was determined by RT-qPCR at 12, 24, and 36 h post-infection (hpi). (**C**) The PABPC1 protein levels were detected at 12, 24, and 36 hpi using antibodies against PABPC1, N, and GAPDH. The PABPC1 level was quantified by ImageJ 1.53c. (**D**) Huh7 cells were transfected with pHA-PABPC1 for 24 h, followed by infection with SADS-CoV at an MOI of 1. Viral replication was determined by Western blotting using a rabbit antibody against the SADS-CoV N protein in-house. (**E**) Huh7 cells were transfected with different doses of pHA-PABPC1 (0, 0.25, 0.5, or 1 µg) and an empty vector for 24 h, and then the cells were infected with SADS-CoV (MOI = 1). The SADS-CoV N protein was determined by Western blotting. (**F**) The N protein levels (green) of SADS-CoV in the presence of PABPC1 (red) were visualized by indirect immunofluorescence. Nuclei were stained with DAPI. Scale bar, 300 µm. (**G**) PABPC1 suppressed viral titers as detected by the TCID50 assay. (**H**) Huh7 cells were transfected with siNC or siPABPC1 (50 µM) for 24 h, followed by infection with SADS-CoV (MOI = 0.1). Western blotting analysis showing upregulation of N protein in the SADS-CoV-infected Huh7 cells with siPABPC1 compared with siNC at 24 and 30 hpi. (**I**) The siNC- and siPABPC1-transfected Huh7 cells were infected with SADS-CoV (MOI = 0.1), and the viral titers were determined by TCID50 assay at 24 hpi. Viral N protein level was quantified by ImageJ 1.53c. Data are shown as the mean ± SD using Student’s *t* test (ns, *p* > 0.05; **, *p* < 0.01; ***, *p* < 0.001). GAPDH was used as a loading control.

PABPs bind to the poly (A) tail of mRNA and promote translation initiation, which is associated with RNA metabolism [13]. Several studies have described that PABPCs are involved in the replication of different DNA or RNA viruses [17,18,19,20,21,22]. However, it is still unclear whether PABPC1, a member of the PABPCs, affects SADS-CoV infection. We detected the transcription or expression level of PABPC1 in SADS-CoV infection at a multiplicity of infection (MOI) of 1. At 12, 24, and 36 h post-infection (hpi), the results of RT-qPCR and Western blotting showed that SADS-CoV infection did not alter PABPC1 RNA (Figure 2A,B) or its protein levels (Figure 2C). To explore the role of PABPC1 in SADS-CoV infection, we initially transfected Huh7 cells with pHA-PABPC1 or an empty vector for 24 h, followed by infection with SADS-CoV at an MOI of 1. The Western blotting results indicated that ectopic expression of PABPC1 remarkably inhibited SADS-CoV N protein expression (Figure 2D). Furthermore, we observed that the viral N protein levels decreased in a dose-dependent manner with increasing PABPC1 expression (Figure 2E). The N protein levels of SADS-CoV in PABPC1-transfected cells were lower than in empty-vector-transfected cells at 24 hpi using IFA (Figure 2F). The results from the TCID50 assay showed that viral titers were significantly decreased in overexpressed PABPC1 cells that were infected with SADS-CoV at an MOI of 1 at 24 hpi (Figure 2G). To further confirm the effect of PABPC1 on SADS-CoV infection, Huh7 cells were transfected with small interfering RNA targeting PABPC1 (siPABPC1) followed by infection with SADS-CoV. The efficiency of SADS-CoV replication was assessed by N protein levels and viral titers using Western blotting and TCID50, respectively. Western blotting results showed that the SADS-CoV N protein level in the siPABPC1-transfected cells was remarkably higher than in the control cells at 24 and 30 hpi during SADS-CoV infection (Figure 2H). Consistent with the result of Western blotting, knockdown of PABPC1 resulted in a remarkable increase in viral titers (Figure 2I). Additionally, porcine PABPC1 also markedly reduced SADS-CoV N protein levels in porcine ileal epithelial (IPI-2I) cells (Figure S2A). These results indicated that PABPC1 inhibits SADS-CoV infection, which serves as a host restriction factor of SADS-CoV replication.

### 3.3. PABPC1 Degrades the N Protein by the Autophagy Pathway

**Figure 3:**
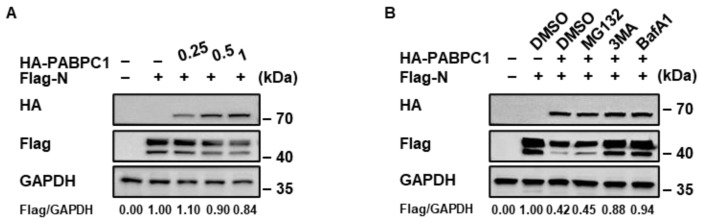
PABPC1 mediates autophagy-lysosomal degradation of the SADS-CoV N protein. (**A**) Different doses of pHA-PABPC1 together with pFlag-N were cotransfected into HEK293T cells. The expression levels of N, PABPC1, and GAPDH were determined by Western blotting using the indicated antibodies. (**B**) HEK293T cells were cotransfected with pFlag-N together with pHA-PABPC1 or an empty vector. After 24 hpt, the cells were treated for 8 h with DMSO, MG132 (20 µM), 3-MA (5 mM), or BafA1 (100 nM), respectively. And then the samples were determined by Western blotting using the indicated antibodies. GAPDH was used as a loading control.

The coronavirus N protein is essential for viral replication because it can form a ribonucleoprotein complex with viral RNA, improve the efficiency of viral RNA transcription, and evade the innate immune response [10,11]. However, the level of N protein was lower in the PABPC1-expressing cells than in the empty-vector-expressing cells, as demonstrated by the cell lysates from our Co-IP assay (Figure 1E). We therefore hypothesized that PABPC1 may restrict SADS-CoV replication by downregulating the abundance of N protein. Different doses of pHA-PABPC1 were cotransfected with pFlag-N into HEK293T cells, and the abundances of N protein were determined by Western blotting. Western blotting indicated that the N protein level was remarkably decreased in the presence of PABPC1, and higher levels of PABPC1 exhibited stronger inhibitory activity (Figure 3A), indicating that PABPC1 may promote N protein degradation. In eukaryotic cells, the ubiquitin-proteasome and autophagy-lysosome pathways are the two major ways of substrate protein degradation. To determine which pathway participates in the degradation of the SADS-CoV N protein mediated by PABPC1, we cotransfected pFlag-N with an empty vector or pHA-PABPC1 into HEK293T cells for 24 h, followed by treatment with either proteasome inhibitor (MG132) or autophagy inhibitors (3-MA or BafA1) for 8 h. Only autophagy inhibitors (3-MA and BafA1), but not MG132, were able to effectively block the reduction in the SADS-CoV N protein caused by PABPC1 (Figure 3B). These results suggested that PABPC1 promotes the degradation of the SADS-CoV N protein through the autophagy-lysosomal degradation pathway.

### 3.4. PABPC1 Promotes TOLLIP-Mediated N Protein Degradation

**Figure 4:**
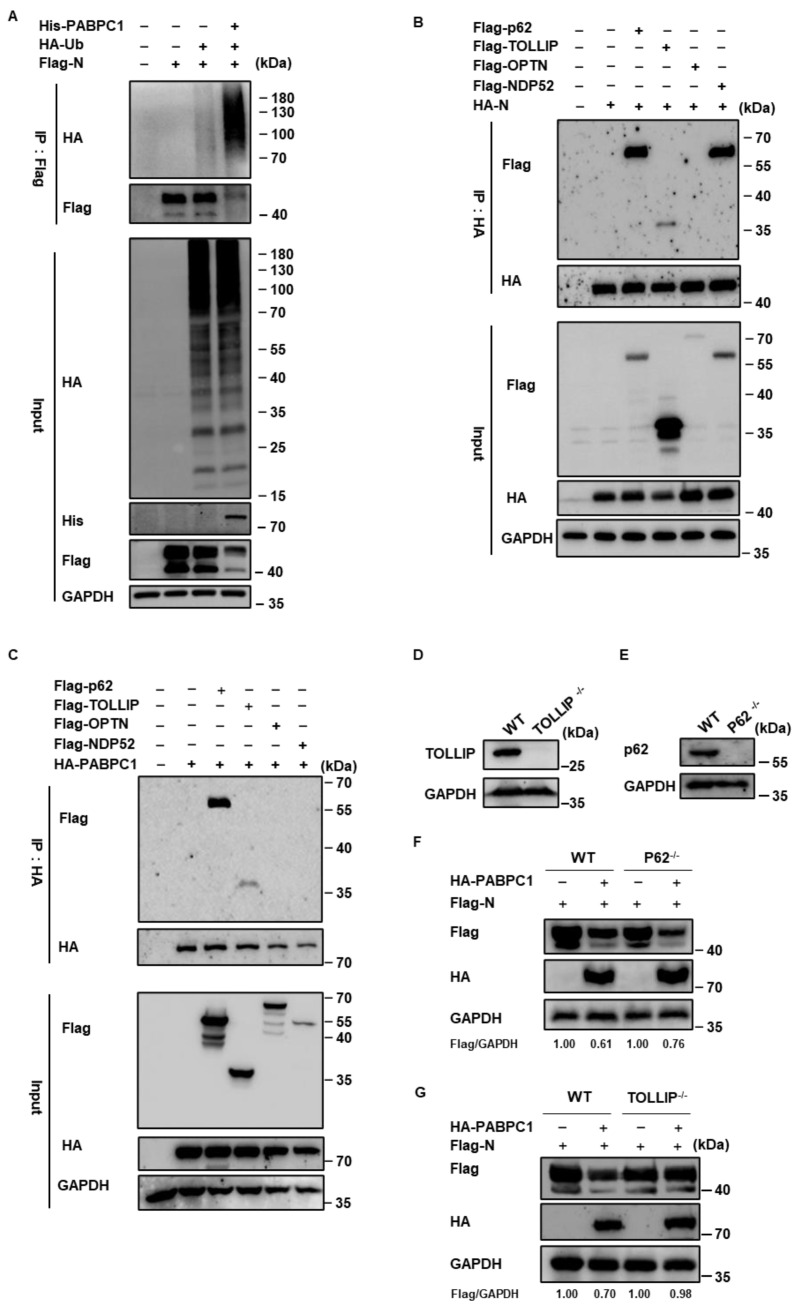
PABPC1 promotes TOLLIP-mediated N protein degradation. (**A**) HEK293T cells were transfected with pHA-Ub, pFlag-N, together with pHis-PABPC1 or an empty vector for 24 h. Cell lysates were immunoprecipitated using Flag-beads, and the ubiquitination of the SADS-CoV N protein was determined by Western blotting. (**B**) HEK293T cells were transfected with pHA-N and Flag-tagged cargo receptors (including p62, TOLLIIP, OPTN, or NDP52). The interactions were determined by Co-IP using HA-beads. (**C**) pFlag-p62, -TOLLIP, -OPTN, or -NDP52 was transfected with pHA-PABPC1 into HEK293T cells. The interactions between cargo receptors and PABPC1 were examined by Co-IP using HA-beads. (**D**) TOLLIP knockout HEK293T cell lines (TOLLIP^−/−^) were validated by Western blotting using anti-TOLLIP antibody. (**E**) Validation of P62 knockout HEK293T cell lines (P62^−/−^) was determined by Western blotting using anti-p62 antibody. (**F**) Wildtype (WT) or P62^−/−^ cells were cotransfected with Flag-N and HA-PABPC1 for 24 h. The expression of N and PABPC1 was detected by immunoblotting. (**G**) WT or TOLLIP^−/−^ cells were transfected with Flag-N and HA-PABPC1 for 24 h, followed by determining the expression of N and PABPC1 by Western blotting. GAPDH was used as a loading control.

Autophagy is a process that recognizes and “eats” non-self or damaged components to maintain intercellular homeostasis. Based on whether autophagy uses selective autophagy receptors (SARs) to recognize cargo and deliver it to the lysosome for degradation, autophagy can be distinguished as selective or non-selective autophagy [25]. Ubiquitin acts as a main “eat-me” signal in the selective autophagic degradation pathway which is recognized by SARs and subsequently delivered to the autophagosome for degradation [25]. Overexpression of PABPC1 significantly enhanced the ubiquitination level of the N protein in HEK293T cells (Figure 4A), indicating that PABPC1 can promote the SADS-CoV N protein ubiquitination. To illustrate the mechanism of PABPC1-mediated selective autophagic degradation of the N protein, we hypothesized that PABPC1 may serve as a bridge between the N protein and those SARs for degradation at the autophagy-lysosome. To determine which SARs are responsible for recognizing and delivering the N protein for autophagic degradation, we examined the interactions between PABPC1, N, and those SARs, including p62/SQSTM1, TOLLIP, OPTN, and NDP52, by Co-IP assay. As shown in Figure 4B, the N protein interacted with p62, TOLLIP, and NDP52. However, we found that PABPC1 only interacted with both p62 and TOLLIP, but not with NDP52 (Figure 4C). We then used the CRISPR/Cas9 method to generate TOLLIP knockout (TOLLIP^−^^/−^) or P62 knockout (P62^−/−^) HEK293T cell lines, respectively, to determine which receptor was necessary for PABPC1-mediated the degradation of the N protein. The expression of TOLLIP or p62 was not detectable in TOLLIP^−/−^ (Figure 4D) or P62^−/−^ (Figure 4E) HEK293T cells, respectively, using the special antibody, indicating that TOLLIP or P62 knockout HEK293T cell lines were successfully established. The wildtype (WT) or knockout HEK293T cells were transfected with pFlag-N along with HA-PABPC1 or an empty vector. We found that knocking out P62 had no impact on the degradation of the N protein mediated by PABPC1 (Figure 4F). In contrast, the expression of N protein was restored in the TOLLIP^-/-^ cells compared with the WT HEK293T cells (Figure 4G). These results showed that TOLLIP, but not p62, functioned as a cargo receptor in the PABPC1-mediated autophagic degradation of the SADS-CoV N protein.

### 3.5. The PABC Domain of PABPC1 Is Critical for the SADS-CoV N Protein Degradation

**Figure 5:**
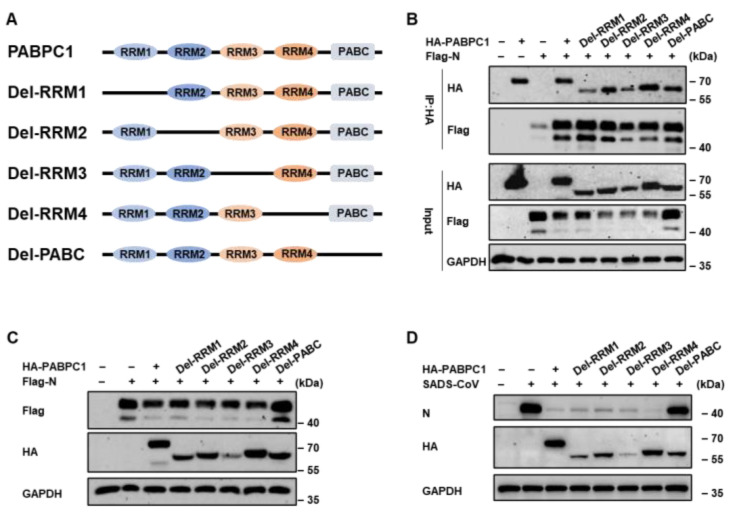
The PABC domain of PABPC1 is essential for restricting SADS-CoV infection. (**A**) Schematic representation of PABPC1 mutants. (**B**) The SADS-CoV N protein associates with the RRM and PABC domains of PABPC1. The plasmids of full-length PABPC1 or its RRM-deletion or PABC-deletion mutants, respectively, were cotransfected with pFlag-N into HEK293T cells for Co-IP assays. Cell lysates were incubated with HA-beads and then subjected to immunoblotting using the indicated antibodies. (**C**) HEK293T cells were cotransfected with pFlag-N and pHA-PABPC1 or its truncated mutants for 24 h. The expression of the N protein was then determined by Western blotting using the indicated antibodies. (**D**) Full-length or truncated mutants of PABPC1 were transfected into Huh7 cells for 24 h, and the cells were infected with SADS-CoV (MOI = 1). Western blotting was used to assess the SADS-CoV replication level using an antibody against the viral N protein at 24 hpi. GAPDH was used as a loading control.

Structurally, PABPC1 harbors four conserved RNA recognition motifs (RRMs) in its N-terminal region and a poly (A)-binding protein C-terminal (PABC) domain in its C-terminal portion. These two regions contribute to RNA recognition and binding, respectively. To delineate the critical domain of PABPC that is responsible for the interaction between PABPC1 and N protein, and the degradation of the SADS-CoV N protein, we constructed several PABPC1 mutants lacking the RRM1, RRM2, RRM3, RRM4, or PABC domains, respectively (Figure 5A). The plasmids of Flag-N and full-length PABPC1 or its mutants were cotransfected into HEK293T cells for Co-IP assay. All mutants of PABPC1 still maintained a significant interaction with N protein (Figure 5B), suggesting that their interaction were not affected by the lack of these individual domains. However, the expression of N protein was significantly restored only in cells expressing the PABPC1 mutant lacking the PABC domain. In contrast, RRM-deletion mutants of PABPC1 did not show this recovery (Figure 5C). Consistently, the results from Western blotting (Figure 5D) showed that the antiviral activity of PABPC1 against SADS-CoV infection in Huh7 cells was lost upon ectopic expression of the PABPC1 PABC-deletion mutant. All of these results demonstrated that SADS-CoV replication is inhibited by the PABC domain of PABPC1, which is a critical region for N protein degradation.

## 4. Discussion

**Figure 6:**
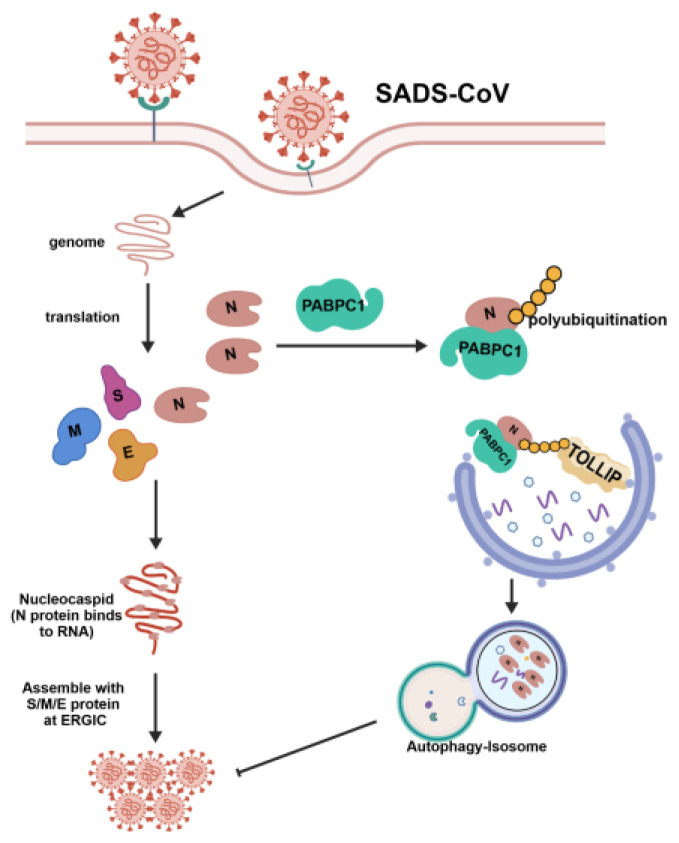
A schematic model of PABPC1 promotes TOLLIP-mediated autophagic degradation of N protein. SADS-CoV enters cells using an unknown receptor and translates its non-structural and structural proteins (S, M, N, and E). These structural proteins cooperate with the viral genome for virion assembly at the endoplasmic-reticulum-to-Golgi intermediate compartment (ERGIC). However, PABPC1 can recruit and degrade the viral N protein through the TOLLIP-mediated autophagic pathway, therefore restricting SADS-CoV replication.

Bats are important reservoir hosts for numerous known or unknown viruses, such as Rabies virus, Nipah virus, SARS-CoV, MERS-CoV, Marburg virus, and Ebola virus, which have caused zoonotic diseases and pose a serious threat to human health [26,27]. SADS-CoV, a novel HKU2-related bat-origin coronavirus, was identified in 2017 and caused fatal disease in piglets in China, indicating that bat-origin coronaviruses have extended their host ranges to pigs and resulted in economic losses in the swine industry [2,3,28]. However, there is no licensed vaccine or therapeutic against SADS-CoV. The coronavirus N protein is an abundantly expressed structural protein that is necessary for RNA recognition and binding, ribonucleocapsid complex packaging, viral RNA synthesis, viral transcription and assembly, and immune evasion [10,11]. All of these functions are essential to coronavirus replication. Characterizing the factors responsible for SADS-CoV infection is therefore necessary to aid the creation of host-directed antiviral therapies. Here, we identified the interactors of the SADS-CoV N protein using immunoprecipitation coupled with mass spectrometry (IP-MS) (Figure 1). We demonstrated that PABPC1 interacts with the N protein and acts as a host restriction factor for SADS-CoV infection by degrading the viral N protein in a TOLLIP-mediated selective autophagic pathway (Figure 6).

Fourteen potential host factors interacting with the SADS-CoV N protein were identified from our IP-MS analysis. These proteins are involved in cytoskeletal regulation, posttranslational modification, RNA processing, and energy production and conversion (Figure 1D). Notably, two homologs of PABPCs (PABPC1 and PABPC4) were observed among the top five hits (Figure 1C), and PABPC4 has been reported to be associated with the coronavirus N protein in previous studies [29,30]. PABPCs are RNA-binding proteins (RBPs) that mainly localize in the cytoplasm and can attach to mRNA to regulate its stability [13]. Previous studies have shown their controversial roles in virus replication, which may be associated with differences between viruses [17,18,19,20,21,22]. PABPC4 exhibits a broad-spectrum antiviral activity for diverse coronaviruses, including SADS-CoV [29,30]. PABPC1 not only has an overall 78% sequence similarity to PABPC4 but also has the highest abundance in our IP-MS data. PABPC1 participates in host translation and disrupts EV-D68 infection, whereas EV-D68 3C^pro^ can cleave PABPC1 to shut off host eukaryotic translation and abrogate its antiviral activity [31]. In addition to its roles in regulating mRNA translation and stability, PABPC1 is also a core component of stress granules (SGs) that are considered a signaling scaffold for the detection of viral RNA by RIG-I-like receptors (RLRs), playing an important role in antiviral responses [16,32]. Our study also indicates PABPC1 as a novel antiviral host factor against SADS-CoV infection. Specifically, overexpression of PABPC1 suppresses viral replication by TOLLIP-mediated selective autophagic degradation of the N protein (Figure 6).

Autophagy is a double-edged sword in virus infection. On the one hand, double-membrane compartments can be formed during the induction of autophagy, protecting viral RNA from recognition and clearance by innate immune sensors. Polioviruses, flaviviruses, coronaviruses, hepatitis C virus, and influenza A virus induce autophagosome formation and accumulation, exploiting them as replication sites to benefit the production of virions [33]. Moreover, autophagy can degrade damaged organelles or lipid droplets to provide energy or metabolites that contribute to viral infection [34]. SADS-CoV not only triggers autophagy but also antagonizes interferon signaling by inducing STAT2 degradation through NBR1- or OPTN- mediated selective autophagy, indicating that SADS-CoV can exploit autophagy to facilitate its replication [35,36]. On the other hand, autophagy can also target virions or viral proteins, a process known as virophagy that is an important antiviral mechanism [34,37]. PABPC4 also exploits the autophagy machinery to degrade the N protein, further restricting SADS-CoV infection [30]. Diverse host restriction factors against coronavirus replication that degrade viral components have been reported in previous studies. Zinc finger protein 219 (ZNF219) and ZNF16 suppress PEDV infection by degrading viral S protein [38,39]. PEDV infection results in a marked upregulation of aldehyde dehydrogenase 1 family member L1 (ALDH1L). However, ALDH1L exerts notable antiviral activity by degrading the viral N and S proteins in a selective autophagy-dependent manner [40]. More importantly, HNRNPA1, a host restriction factor against PEDV infection, degrades viral N protein to facilitate the host antiviral response. Conversely, PEDV N protein also antagonizes the restrictive function of HNRNPA1 by promoting its degradation [41]. We also determined whether SADS-CoV infection regulates PABPC1 expression. No significant difference in PABPC1 levels was observed in SADS-CoV infection, which excludes that the viral infection can disrupt PABPC1 expression to evade its antiviral activity (Figure 2A–C). SADS-CoV is a bat-originated coronavirus [3], and PABPC1 from humans, pigs, and bats shares almost 100% sequence identity. We thus hypothesized that bat PABPC1 may exert comparable antiviral activity against SADS-CoV.

In the selective autophagy process, ubiquitin modification of cargo mediated by E3 ubiquitin ligases and recognition by SARs are essential for autophagic degradation. Our study verified that PABPC1 promotes the ubiquitination of the N protein and found that TOLLIP is the primary cargo receptor responsible for autophagic degradation of the SADS-CoV N protein (Figure 4). However, PABPC1 is not an E3 ubiquitin ligase, indicating that it may recruit an E3 ubiquitin ligase to attach the ubiquitin chains to the N protein for TOLLIP-mediated autophagic degradation. Interestingly, while all PABPC1 mutants can interact with the SADS-CoV N protein, only the PABC-deletion mutant lost the capacity of PABPC1 to mediate N protein degradation and restrict SADS-CoV infection. These results indicate that all of these domains are required for the interaction between PABPC1 and N protein, and that when one of the domains of PABPC1 is absent, the other domains may compensate for the loss of its function in their interaction.

SADS-CoV has only been detected in pigs, but it can infect more than 20 mammalian cells, including those of humans, bats, monkeys, and pigs, implying that SADS-CoV may further extend its host range [4,5]. SADSr-CoVs share a high degree of genome size and S protein sequence similarity with SADS-CoV, have been detected in bats, and show potential for interspecies infection [3,9]. The major differences between SADS-CoV and SADSr-CoVs are in the S, NS7a, and NS7b proteins [9]. Over 96% of sequences of the N protein of SADS-CoV and SADSr-CoVs are similar, indicating that PABPC1 may have antiviral activity against SADSr-CoVs through the same antiviral mechanism that it uses against SADS-CoV. It is a topic worthy of further exploration.

## 5. Conclusions

Our research identifies PABPC1 as a host restriction factor responsible for SADS-CoV N protein degradation. Mechanistically, PABPC1 interacts with the SADS-CoV N protein and promotes its degradation; the PABC domain of PABPC1 is critical for this process, which limits SADS-CoV infection through TOLLIP-mediated selective autophagy. The study not only contributes to improving our knowledge of the SADS-CoV–host interaction but also highlights virophagy as a possible antiviral therapeutic strategy and provides additional antiviral targets for infection with SADS-CoV and SADS-related CoVs.

## Figures and Tables

**Table 1:** Primers used in the study.

Primers	Sequence (5′–3′)
His-PABPC1-F	ATTTCCGGTGAATTCATGAACCCCAGCGCC
His-PABPC1-R	AGAGGGGCGGGATCCCTAGTGGTGGTGGTGGTGGTGGCCGCCAACAGTTGGAACACC
HA-PABPC1-F	GATTACGCTGAATTCATGAACCCCAGTGCC
HA-PABPC1-R	ATCTGCTAGCTCGAGTTAAACAGTTGGAAC
HA-PABPC1-del-RRM1-F	GATTACGCTGAATTCATGAACCCCAGTGCCCCCAGCTACCCCATGGCCTGGTCTCAGCGTGAT
HA-PABPC1-del-RRM2-F	AAAAGTGGAGTAGGCCGATTTAAGTCTCGT
HA-PABPC1-del-RRM2-R	ACGAGACTTAAATCGGCCTACTCCACTTTT
HA-PABPC1-del-RRM3-F	GCAAAAGAATTCACCCGAGCTCAGAAAAAG
HA-PABPC1-del-RRM3-R	CTTTTTCTGAGCTCGGGTGAATTCTTTTGC
HA-PABPC1-del-RRM4-F	AGATACCAGGGTGTTTTAGCTCAGCGCAAA
HA-PABPC1-del-RRM4-R	TTTGCGCTGAGCTAAAACACCCTGGTATCT
HA-PABPC1-del-PABC-F	TGACTGCTTCCATGTTGGCATCTGCCCCAAGCTAAAGAGGCT
HA-PABPC1-del-PABC-R	AGCCTCTTTAGCTTGGGGCAGATGCCAACA
qGAPDH-F	GGAGCGAGATCCCTCCAAAAT
qGAPDH-R	GGCTGTTGTCATACTTCTCATGG
qSADS-CoV-F	CTAAAACTAGCCCCACAGGTC
qSADS-CoV-R	TGATTGCGAGAACGAGACTG

## Data Availability

All original findings of this study are presented in the Materials and Methods Section. Further inquiries should be addressed to the corresponding authors.

## Supplementary Materials

The following supporting information can be downloaded at: https://www.mdpi.com/article/10.3390/pathogens15070752/s1, Figure S1: PABPC1 interacts with the SADS-CoV N protein in an RNA-dependent manner; Figure S2: Porcine PABPC1 inhibits SADS-CoV replication.

## References

[1] Cui J., Li F., Shi Z.L. (2019). Origin and evolution of pathogenic coronaviruses. Nat. Rev. Microbiol..

[2] Pan Y., Tian X., Qin P., Wang B., Zhao P., Yang Y.L., Wang L., Wang D., Song Y., Zhang X. (2017). Discovery of a novel swine enteric alphacoronavirus (SeACoV) in southern China. Vet. Microbiol..

[3] Zhou P., Fan H., Lan T., Yang X.L., Shi W.F., Zhang W., Zhu Y., Zhang Y.W., Xie Q.M., Mani S. (2018). Fatal swine acute diarrhoea syndrome caused by an HKU2-related coronavirus of bat origin. Nature.

[4] Yang Y.L., Qin P., Wang B., Liu Y., Xu G.H., Peng L., Zhou J., Zhu S.J., Huang Y.W. (2019). Broad cross-species infection of cultured cells by bat HKU2-related swine acute diarrhea syndrome coronavirus and identification of its replication in murine dendritic cells in vivo highlight its potential for diverse interspecies transmission. J. Virol..

[5] Edwards E.E., Yount B.L., Graham R.L., Leist S.R., Hou Y.J., Dinnon K.H., Sims A.C., Swanstrom J., Gully K., Scobey T.D. (2020). Swine acute diarrhea syndrome coronavirus replication in primary human cells reveals potential susceptibility to infection. Proc. Natl. Acad. Sci. USA.

[6] Luo Y., Chen Y., Geng R., Li B., Chen J., Zhao K., Zheng X.S., Zhang W., Zhou P., Yang X.L. (2021). Broad cell tropism of SADS-CoV in vitro implies its potential cross-species infection risk. Virol. Sin..

[7] Chen Y., Jiang R.D., Wang Q., Luo Y., Liu M.Q., Zhu Y., Liu X., He Y.T., Zhou P., Yang X.L. (2022). Lethal swine acute diarrhea syndrome coronavirus infection in suckling mice. J. Virol..

[8] Duan Y., Yuan C., Suo X., Li Y., Shi L., Cao L., Kong X., Zhang Y., Zheng H., Wang Q. (2023). Bat-origin swine acute diarrhea syndrome coronavirus is lethal to neonatal mice. J. Virol..

[9] Luo Y., Gong Q.C., Yao Y.L., Chen Y., Si H.R., Xie X., Yang Y.L., Feng Z.X., Jiang R.D., Huang Y.W. (2025). Swine acute diarrhea syndrome coronavirus-related viruses from bats show potential interspecies infection. J. Virol..

[10] Bai C., Zhong Q., Gao G.F. (2022). Overview of SARS-CoV-2 genome-encoded proteins. Sci. China Life Sci..

[11] Bai Z., Cao Y., Liu W., Li J. (2021). The SARS-CoV-2 nucleocapsid protein and its role in viral structure, biological functions, and a potential target for drug or vaccine mitigation. Viruses.

[12] Craig A.W., Haghighat A., Yu A.T., Sonenberg N. (1998). Interaction of polyadenylate-binding protein with the eIF4G homologue PAIP enhances translation. Nature.

[13] Mangus D.A., Evans M.C., Jacobson A. (2003). Poly(A)-binding proteins: Multifunctional scaffolds for the post-transcriptional control of gene expression. Genome Biol..

[14] Yi H., Park J., Ha M., Lim J., Chang H., Kim V.N. (2018). PABP cooperates with the CCR4-NOT complex to promote mRNA deadenylation and block precocious decay. Mol. Cell.

[15] Bresson S., Tollervey D. (2018). Tailing Off: PABP and CNOT Generate Cycles of mRNA Deadenylation. Mol. Cell.

[16] Jain S., Wheeler J.R., Walters R.W., Agrawal A., Barsic A., Parker R. (2016). ATPase-modulated stress granules contain a diverse proteome and substructure. Cell.

[17] Wu T., Wei X., Zheng S., She G., Han Z., Xu Z., Cao Y., Xue C. (2022). Poly(A)-binding protein cytoplasmic 1 inhibits porcine epidemic diarrhea virus replication by interacting with nucleocapsid protein. Viruses.

[18] Grizer C.S., Messacar K., Mattapallil J.J. (2024). Enterovirus-D68—A reemerging non-polio enterovirus that causes severe respiratory and neurological disease in children. Front. Virol..

[19] Davis D.V., Choi E.J., Ismail D., Hernandez M.L., Choi J.M., Zhang K., Khatkar K., Jung S.Y., Wu W., Bao X. (2024). Role of poly(a)-binding protein cytoplasmic 1, a tRNA-derived RNA fragment-bound protein, in respiratory syncytial virus infection. Pathogens.

[20] Bouillier C., Cosentino G., Léger T., Rincheval V., Richard C.A., Desquesnes A., Sitterlin D., Blouquit-Laye S., Eléouët J.F., Gault E. (2019). The interactome analysis of the respiratory syncytial virus protein M2-1 suggests a new role in viral mRNA metabolism post-transcription. Sci. Rep..

[21] Zhai H., Qin W., Dong S., Yang X., Zhai X., Tong W., Liu C., Zheng H., Yu H., Kong N. (2023). PEDV N protein capture protein translation element PABPC1 and eIF4F to promote viral replication. Vet. Microbiol..

[22] Wang J., Zhang X.Z., Sun X.Y., Tian W.J., Wang X.J. (2024). Cellular RNA-binding proteins LARP4 and PABPC1 synergistically facilitate viral translation of coronavirus PEDV. Vet. Microbiol..

[23] Xu Z., Zhang Y., Gong L., Huang L., Lin Y., Qin J., Du Y., Zhou Q., Xue C., Cao Y. (2019). Isolation and characterization of a highly pathogenic strain of porcine enteric alphacoronavirus causing watery diarrhoea and high mortality in newborn piglets. Transbound. Emerg. Dis..

[24] Yuan C., Suo X., Duan Y., Li X., Shi L., Cao L., Kong X., Zheng H., Wang Q. (2022). Comprehensive subcellular localization of swine acute diarrhea syndrome coronavirus proteins. J. Virol..

[25] Lamark T., Johansen T. (2021). Mechanisms of Selective Autophagy. Annu. Rev. Cell Dev. Biol..

[26] Zhou S., Liu B., Han Y., Wang Y., Chen L., Wu Z., Yang J. (2022). ZOVER: The database of zoonotic and vector-borne viruses. Nucleic Acids Res..

[27] Letko M., Seifert S.N., Olival K.J., Plowright R.K., Munster V.J. (2020). Bat-borne virus diversity, spillover and emergence. Nat. Rev. Microbiol..

[28] Wang L., Su S., Bi Y., Wong G., Gao G.F. (2018). Bat-origin coronaviruses expand their host range to pigs. Trends Microbiol..

[29] Jiao Y., Kong N., Wang H., Sun D., Dong S., Chen X., Zheng H., Tong W., Yu H., Yu L. (2021). PABPC4 Broadly Inhibits Coronavirus Replication by Degrading Nucleocapsid Protein through Selective Autophagy. Microbiol. Spectr..

[30] Zhao C., Qin Y., Huang H., Chen W., Hu Y., Zhang X., Li Y., Lan T., Sun W. (2025). PABPC4 inhibits SADS-CoV replication by degrading the nucleocapsid protein through selective autophagy. Vet. Sci..

[31] Tan R., Zhang Y., Huang M., Chen H., Liu Z., Wang Z., Li X., Wang T., Wang Z. (2025). EV-D68 cleaves LARP1 and PABPC1 by 3Cpro to redirect host mRNA translation machinery toward its genomic RNA. PLoS Pathog..

[32] Onomoto K., Yoneyama M., Fung G., Kato H., Fujita T. (2014). Antiviral innate immunity and stress granule responses. Trends Immunol..

[33] Choi Y., Bowman J.W., Jung J.U. (2018). Autophagy during viral infection—A double-edged sword. Nat. Rev. Microbiol..

[34] Mijaljica D., Klionsky D.J. (2020). Autophagy/virophagy: A “disposal strategy” to combat COVID-19. Autophagy.

[35] Shi D., Zhou L., Shi H., Zhang J., Zhang J., Zhang L., Liu D., Feng T., Zeng M., Chen J. (2023). Autophagy is induced by swine acute diarrhea syndrome coronavirus through the cellular IRE1-JNK-Beclin 1 signaling pathway after an interaction of viral membrane-associated papain-like protease and GRP78. PLoS Pathog..

[36] Zeng S., Zhao Y., Peng O., Xia Y., Xu Q., Li H., Xue C., Cao Y., Zhang H. (2022). Swine acute diarrhea syndrome coronavirus induces autophagy to promote its replication via the Akt/mTOR pathway. iScience.

[37] Chen T., Tu S., Ding L., Jin M., Chen H., Zhou H. (2023). The role of autophagy in viral infections. J. Biomed. Sci..

[38] Zhao W., Kong N., Lan D., Zhang Y., Liu H., Liu C., Qin W., Yang X., Liu T., Tong W. (2026). ZNF219 inhibits porcine epidemic diarrhea virus by degrading viral S2 protein. Virol. Sin..

[39] Zheng D., Yang X., Qin W., Gao A., Liu Y., Sun H., Tong W., Yu H., Zheng H., Tong G. (2026). ZNF16 inhibits PEDV replication through autophagy-mediated degradation of S1 protein. Vet. Microbiol..

[40] Wang J., Zeng Y., Liu Y., Sun H., Gao A., Zheng D., Tong W., Yu H., Zheng H., Tong G. (2026). ALDH1L1 suppresses the replication of porcine epidemic diarrhea virus by degrading viral nucleocapsid and envelope proteins. J. Virol..

[41] Zhai X., Kong N., Zhang Y., Song Y., Qin W., Yang X., Ye C., Ye M., Tong W., Liu C. (2023). N protein of PEDV plays chess game with host proteins by selective autophagy. Autophagy.

