# Supplementary material for: PABPC1 Restricts Bat-Origin Swine Acute Diarrhea Syndrome Coronavirus Infection via TOLLIP-Mediated Degradation of Viral Nucleocapsid Protein
Source: Pathogens. 2026 Jul 17;15(7):752. doi: 10.3390/pathogens15070752 (PMC13416350; doi:10.3390/pathogens15070752)

PABPC1 Restricts Bat-Origin Swine Acute Diarrhea Syndrome Coronavirus Infection via TOLLIP-Mediated Degradation of Viral Nucleocapsid Protein

Maowen Sun, Cong Yuan, Yu Zhang, Xueliang Zhu, Lei Shi, Yueyue Duan, Wenquan Mao, Luyao Li, Yanghe Liu and Qi Wang *

State Key Laboratory of Animal Disease Control and Prevention, Lanzhou Veterinary Research Institute, Chinese Academy of Agricultural Sciences, Lanzhou 730046, China
* Correspondence: qiwang@caas.cn

Supplementary information

Figure S1. PABPC1 interacts with the SADS-CoV N protein in an RNA-dependent manner. (A) HEK293T cells were transfected with pFlag-N and pHA-PABPC1, cell lysate was treated with or without RNase at 37℃ for 30 min, followed by incubated with Flag-beads overnight at 4℃. The protein samples were used to Western blotting using the indicated antibodies.

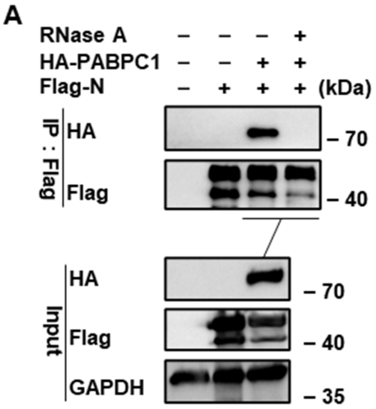

Figure S2. Porcine PABPC1 inhibits SADS-CoV replication. (A) Porcine ileal epithelial (IPI-2I) cells were transfected with the plasmid of porcine PABPC1 (pPABPC1) for 24 h, using human PABPC1 (hPABPC1) as a control, followed by infection with SADS-CoV (MOI = 1). The N protein levels were determined by western blotting using an anti-N antibody. GAPDH served as loading control.

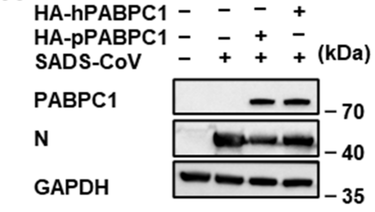

Supplement: Supplementary file 1 [file pathogens-15-00752-s001.zip › pathogens-4304837-supplementary.pdf]
